# Twin Gestation in a Septate Bicornuate Uterus

**DOI:** 10.1155/2012/563085

**Published:** 2012-12-04

**Authors:** Henry Osazuwa, David Ejenobo

**Affiliations:** Maternity Unit, Nisa Premier Hospital, Plot 115–117, Alex Ekwueme Street, P.O. Box 7320, Jabi, Abuja 900001, Nigeria

## Abstract

Bicornuate and septate uteri are among the commonest Mullerian anomalies. They are sporadic and fairly distinguished, but hybrid deformities can occur. This combination creates aetiological and clinical difficulties. The alternative theory of concurrent fusion and septal resorption of the Mullerian duct is seen as the basis of the altered foetal embryology, while the favourable outlook of a bicornuate uterus may be offset by the suboptimal implantation across the avascular septum. Obstetric care is based on empirical interventions deduced from case series, with varied and inconsistent outcomes. We present a 32-year-old primipara with a dizygotic twin gestation in separate compartments of a septate bicornuate uterus. She had an elective bilateral caesarean delivery at term with an accidental septal resection for morbidly adherent placenta. Although a summation of obstetric risks is a possibility, an excellent outcome was observed.

## 1. Introduction

Mullerian anomalies may be relatively uncommon, but the disproportionate interest in these clinical entities is due to their link to a myriad of poor pregnancy outcomes, which include miscarriages, preterm deliveries, malpresentation, prelabour rupture of membranes, intrauterine growth restriction, postpartum haemorrhage, and retained placenta [1]. Previous attempts at defining a population-based prevalence had proved daunting because they are either asymptomatic or the uterine cavity is competent enough to accommodate normal foetal growth and development. Notwithstanding, recent estimates put congenital uterine anomalies in an unselected population at 5.5%, with septate/subseptate and bicornuate uteri contributing 2.3% and 0.4%, respectively. In women with recurrent miscarriages or subfertility, these figures maybe higher [2, 3]. The underlying trigger for interruptions in the development of the Mullerian ducts at key stages in foetal embryology is multifactorial, encompassing genetic, environmental, and pharmacologic factors [4]. Chromosomal damage can result from exposure to ionization radiation, as well as intrauterine infections, like rubella. In the past, thalidomide and diethylstilbestrol caused limb and genital tract defects, but these agents are mentioned now for historical references.

In the early intrauterine life, between 6th and 12th weeks of pregnancy, the paramesonephric (Mullerian) ducts in their caudal end fuse with the Y-shaped tubular uterovaginal primordium, which later forms the uterus and vagina. The uterine segment fuses and subsequently becomes canalized with regression of the central septum through a process of apoptosis regulated by the Bcl-2 gene. This development occurs mainly in sequence, as shown by the variation in observed anomalies, but concurrent lateral fusion and septal resorption have also been suggested [5, 6]. The American Fertility Society describes morphological categories, which are widely used for clinical management and with respect to this paper two are relevant. The bicornuate uterus is included in the class IV (fusion) defects, where there is a partial fusion of the Mullerian ducts. There is typically a deep fundal indentation >1 cm between the horns, with a central myometrium covered by endometrium, which may extend to the level of the internal os. The key here is the endometrial covering. The other category is the class V (Canalization) defect, represented by a septate uterus, in which there is a failure of resorption of the septum between the two uterine horns and can be partial or complete. The septum is mainly muscular superiorly, histologically different from the normal myometrium, and more fibrous and thinner towards the cervix. The fundus maybe convex, flat, or with a concavity that is <1 cm [7, 8]. 

This paper involves a defect which appears to represent a composite anomaly involving a combination of a bicornuate uterus and a septate uterus, supporting the hypothesis that lateral fusion and canalization may occur concurrently. When uterine anomalies are first recognized in pregnancy, they create a series of clinical dilemmas because of their reputation for miscarriages, preterm delivery, and surgical interventions. Abnormal uterine anatomy predisposes to malpresentation and related vascular aberrations involving the uterine or ovarian arteries may cause intrauterine growth restriction. As such we highlight empirical antenatal interventions and an accidental surgical resection of a septum at caesarean delivery. 

## 2. Case Presentation

The patient is a 32-year-old para 1^+0^ who had an uneventful spontaneous vaginal delivery in 2009. She was first seen at 7 weeks of gestational age with complaints of scanty bleeding per vagina, which had persisted for a week. There was no abdominal pain, dizziness, or fainting spells. A trans-vaginal ultrasound scan to localize and ascertain foetal viability revealed a uterus that appeared bicornuate, with an intervening septum, containing two foetuses in separate sacs, which showed normal cardiac activities. The crown-rump lengths of 0.68 cm for both babies corresponded to a gestational age of 6 weeks and 4 days. A subchorionic haematoma (diameter of 0.6 cm) was noticed beneath the placenta in the right cornum, which resolved spontaneously over 4 weeks. Luteal cysts were seen in both ovaries. She was admitted for bed rest and placed on cyclogest (progesterone) vaginal pessary, 400 mg daily until 10 weeks. Her hematocrit was 35%, blood group was O rhesus positive, with a Negative Venereal Diseases Research Laboratory test, hepatitis B surface antigen, and retroviral screen. She was allowed home after 4 days.

**Figure 1:**
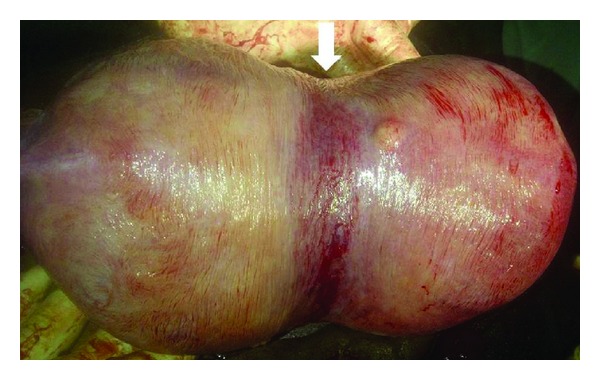
Bicornuate morphology with the typical fundal indentation (arrow).

**Figure 2:**
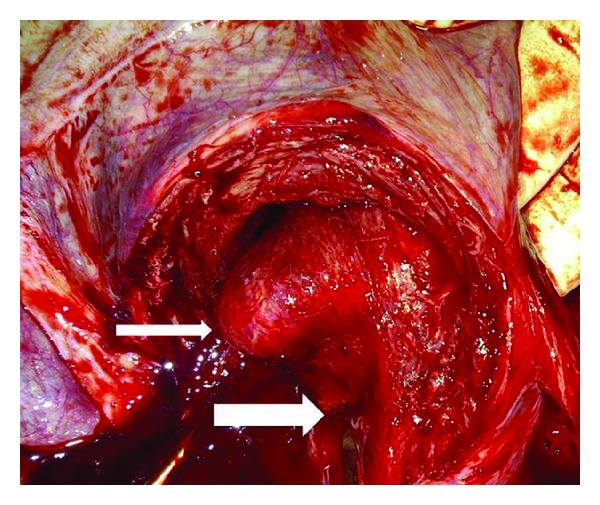
Dividing septum with a finger from the adjacent cavity (small arrow). Communication between the inferior edge of the septum and the internal cervical os (large arrow).

A prophylactic cerclage was inserted at 13 weeks based on the uterine anomaly and twin gestation, using the McDonald technique which was knotted at 12°clock. A detailed anomaly scan was performed at 20 weeks, and subsequent follow-up ultrasound scans at 28, 32, and 36 weeks showed two foetuses in separate compartments with a dividing septum. Liquor volume estimates were normal as well as foetal biometric parameters, which were consistently compatible with respective gestational ages. Two courses of intramuscular dexamethasone, 24 mg in two divided doses, were administered at 28 and 32 weeks. The distorted bicornuate uterine morphology was obvious on inspection of the abdomen in the third trimester. An elective caesarean delivery was performed at 37 weeks under subarachnoid block. The initial Monroe Kerr incision entered mainly the right cavity through which the first twin was delivered. A broad septum prevented access to the second cavity, prompting the placement of another incision to deliver the second twin. A bicornuate uterus (Figure 1) was noted with a central fibrous septum (Figure 2), devoid of a covering of endometrial tissue (confirmed by histology), extending to a finger's breadth above the cervix. The foetal outcomes were male and female neonates, weighing 3.0 kg and 2.5 kg, respectively, with APGAR scores of 8 in 1 minute and 10 in 5 minutes.

While the left placenta was delivered easily by controlled cord traction, there was difficulty in delivering the right placenta because of significant placental infiltration through the fundal aspect of the septum, with haemorrhage from the partially separated segment. This necessitated a vertical, approximately 6 cm uterine incision, upwards from the original incision, which created enough room for removal of the septum up to its base, with four haemostatic figures of eight stitches applied along the base. The final inverted “T” incision was closed in three layers. She was discharged home after 5 days, and her postnatal period was uneventful. The need for a repeat caesarean delivery at subsequent births was explained.

## 3. Discussion

A prior uneventful pregnancy in obstetric settings is often viewed as a good indicator of subsequent outcomes, so the diagnosis of a major uterine malformation in the next pregnancy is almost always a profound finding, particularly in addition to twin gestation. Ultrasound scan remains the sole and reliable means of assessing the presence of these anomalies in pregnancy for safety reasons, as radiation and invasive diagnostic tools can jeopardise a viable intrauterine pregnancy. The core morphological features are displayed properly in the first trimester. On transabdominal scan (TAS), a septate uterus appears as two cavies without the sagittal notching and without the fundal myometrium. TAS also allows for urological assessment, while 3D imaging enhances the accuracy of the diagnosis. In this patient, the early ultrasound scan confirmed the presence of uterine anomaly, in favour of a bicornuate uterus. The placement of a cervical cerclage is an acceptable compromise when the integrity of the cervix is in doubt, as in Mullerian anomalies. The incompetence of the cervix is an imprecise definition because it involves both a structural as well as the functional defect, which unlike the formal, may show no physical evidence until uterine evacuation is terminal. A cervical cerclage was placed using the McDonald's technique at 13 weeks, as an adjunctive procedure to minimize the risk of a miscarriage or preterm birth [9].

Preemptive foetal lung maturation is a useful option when there is a risk for preterm delivery as in this paper. In the foetal lungs, corticosteroid leads to increase in protein production, biosynthesis of phospholipids, and the appearance of surfactants, reducing the risk of respiratory distress syndrome, intraventricular haemorrhage, and necrotizing enterocolitis [10]. The dexamethasone given at 28 and 32 weeks was aimed at achieving these advantages, though debate is ongoing as to the impact and long-term effect of the second dose of corticosteroid. In septate uterus, the resection of the intervening septum is often reserved for cases of recurrent miscarriages and preterm delivery, which can conveniently be excised hysteroscopically outside pregnancy, but an accidental septal resection was inevitable because of haemorrhage from the morbidly implanted placenta [8]. A bilateral caesarean section is the norm when there is a uterine septum extending to the lower segment. Important lesson here is that a skilled professional should attend these operative deliveries because unexpected complications may arise and ultrasonographic evaluation, particularly 2D, may not be detailed enough to give precise morphological definition. Also a previous uneventful pregnancy does not eliminate the presence of a significant congenital anomaly. Although karyotyping was not done, the discordant sex of the babies inferred a dizygotic twin. This paper demonstrates a favourable outcome for the mother and baby, achieved by applying relatively basic obstetric interventions.

## 4. Conclusion

Congenital Mullerian anomalies are often sporadic, and rarely hybrid deformities can occur. Basic obstetric interventions may be enough to achieve optimal care in the presence of significant defects, and notably a previous normal childbirth does not exclude the presence of major anomalies.
